# Platelet-Rich Plasma for Knee Osteoarthritis and Patient-Reported Outcomes: A Large Retrospective Analysis

**DOI:** 10.1177/19417381261496039

**Published:** 2026-10-26

**Authors:** Jonathan P. Coss, Kenar Z. Gelman, Ryan Lingor, Kaylre Greaves, Courtney Melone, Michael Parides, Brian C. Halpern

**Affiliations:** † Department of Primary Care Sports Medicine, Hospital for Special Surgery, New York, New York

**Keywords:** knee, knee osteoarthritis, platelet-rich plasma (PRP)

## Abstract

**Background:**

Platelet-rich plasma (PRP) is used increasingly to treat knee osteoarthritis (OA). This study aimed to describe PRP use for knee OA and identify patient- and treatment-specific factors associated with clinical outcomes.

**Hypothesis:**

Patients with knee OA undergoing intra-articular PRP injections would demonstrate improvement in pain, function, and symptoms over time, with outcomes varying by patient and treatment characteristics.

**Study Design:**

Retrospective cohort study.

**Level of Evidence:**

Level 3.

**Methods:**

A total of 743 patients with knee OA were treated with intra-articular PRP injections. Outcomes were assessed using the Numeric Rating Scale (NRS, 0-100, higher scores indicating greater pain) and the Knee Injury and Osteoarthritis Outcome Score for Joint Replacement (KOOS-JR). Change in patient-reported outcome measure (PROM) scores from baseline and achievement of patient acceptable symptom state (PASS) were measured. Repeated-measures mixed models evaluated outcomes at baseline, 6 months, and 1 year using an all-response analysis (391 patients for Pain NRS; 400 for KOOS-JR).

**Results:**

PRP injections were associated with improvement in pain, function, and symptom state. Pain NRS improved from 39.2 at baseline to 27.2 at 6 months and 22.4 at 1 year (both *P* < 0.001), while KOOS-JR improved from 60.7 to 68.5 and 69.1 (both *P* < 0.001), with similar findings in the complete-case cohort. When stratified by radiographic severity, patients with Kellgren-Lawrence (KL) grades 1 to 3 improved in both outcomes at 6 months and 1 year, while patients with KL grade 4 improved in Pain NRS at 1 year (*P* < 0.001) and KOOS-JR at 6 months (*P* = 0.01) and 1 year (*P* = 0.001). PASS achievement rates increased over time for both pain and function.

**Conclusion:**

Intra-articular PRP injections were associated with improvements in pain, function, and symptoms in patients with knee OA. Differences between complete-case and all-response analyses suggest that follow-up patterns may influence estimated treatment response.

**Clinical Relevance:**

These findings describe patient-reported outcomes after intra-articular PRP injection for knee OA. Because this study lacked a control group, findings should not be interpreted as evidence that PRP caused the observed improvements.

Knee osteoarthritis (OA) is a leading cause of pain, functional limitation, and disability worldwide, particularly among middle-aged and older adults.^19,28,30^ Worldwide, >364 million people are affected by knee OA, highlighting the growing need for safe and effective treatment options.^11,38^ Patients commonly present with progressive knee pain, reduced activity tolerance, and impaired quality of life.^11,28^ First-line management typically involves conservative measures such as weight loss, physical therapy, activity modification, oral medications, chondroprotective agents, and intra-articular corticosteroid injections.^3,22,23,26^ These treatments are intended primarily to provide symptomatic relief and are often associated with only short-term benefit.^27^ In more advanced cases, surgical interventions may be considered.^26^

Platelet-rich plasma (PRP) has emerged as a biologic treatment option intended to improve symptoms through modulation of inflammation and the delivery of growth factors.^1,18^ PRP is an autologous blood-derived product with a high concentration of platelets prepared from a patient’s own blood. By delivering concentrated growth factors to the affected area, PRP is thought to enhance tissue repair and may potentially improve symptoms in OA.^17^ Despite increasing clinical use, the efficacy of PRP for knee OA remains debated.^4,12,29^ Randomized controlled trials have reported conflicting results, and variability in PRP preparation, injection technique, and patient selection has limited the reproducibility and interpretation of treatment efficacy.^9,15,24^

Growing evidence suggests that both patient-specific and procedural factors may influence the clinical response to PRP therapy.^16,32^ However, standardized reporting of demographic, radiographic, and treatment variables remains limited,^9,33^ and predictors of treatment success are not well defined.^4,5^ Improved characterization of these factors may help optimize patient selection and treatment delivery. Therefore, a large-scale retrospective study was undertaken to better define the use of PRP in patients with knee OA and to evaluate patient- and treatment-specific factors associated with clinical outcomes.

The purpose of this study was to characterize the use of intra-articular PRP injections for knee osteoarthritis and to evaluate whether patient and treatment characteristics are associated with clinical outcomes. This study had 2 primary objectives: first, to characterize the historical use of PRP for knee OA at our institution with respect to both patient demographics and treatment factors; and second, to determine the extent to which these factors were associated with treatment success, defined by reduction in knee pain, improvement in function, and patient-reported symptom state. We hypothesized that patients with knee OA undergoing intra-articular PRP injections would demonstrate improvements in pain and function over time, with outcomes varying according to patient and injection characteristics.

## Methods

This study was approved by the institutional review board (IRB) at the Hospital for Special Surgery (HSS) (No. 20240782). The investigation followed accepted reporting standards for observational studies.

Patients who underwent standalone intra-articular PRP injections performed by 2 fellowship-trained sports medicine physicians between August 30, 2020 and December 31, 2024 were extracted from the Center for Regenerative Medicine Registry at the HSS and screened for eligibility. Patient data were collected with IRB approval (HSS IRB no. 2020-0241). Arthrex ACP (autologous conditioned plasma) system was utilized; after centrifugation, 4 ml of PRP was injected via an anterolateral approach.

Inclusion criteria consisted of patients with a diagnosis of knee OA treated with intra-articular PRP injection during the study period. A total of 743 patients underwent PRP injection and constituted the treated cohort; of these, 405 patients contributed data to ≥1 patient-reported outcome measures (PROM) analysis, including 391 patients for Numeric Rating Scale for Pain (Pain NRS) and 400 patients for Knee Injury and Osteoarthritis Outcome Score for Joint Replacement (KOOS-JR) (see Table 1 for time-specific sample sizes at baseline, 6 months, and 1 year). Demographic and clinical variables collected included age, sex, race and ethnicity, medication use at the time of injection, knee laterality, and Kellgren-Lawrence (KL) radiographic grade.^21^ Procedural variables were extracted from the clinical record, including PRP preparation kit, leukocyte composition, aspirate source and volume, final injectate volume, injection approach, needle size, ultrasound (US) guidance, aspiration of joint fluid, anesthetic use and delivery method, and postprocedure care protocols. All injections utilized leukocyte-poor PRP preparations. Patients were instructed to avoid nonsteroidal anti-inflammatory drugs (NSAIDs) for 1 week before and 1 week after injection. No formal physical therapy protocol was prescribed; instead, patients were provided with a home exercise program consisting of quadriceps sets and straight leg raises. The all-response analysis of patient enrollment and response availability is presented in Table 1, which reports sample sizes for Pain NRS and KOOS-JR separately at each of the 3 timepoints (baseline, 6 months, 1 year).

**Table 1.** All-response analysis of patient enrollment and response availability for baseline, 6-month, and 1-year timepoints, among the 743 patients in the treated cohort

Treated cohort (N = 743)	Timepoint	Pain NRS, n	KOOS-JR, n
	Baseline	342	343
	6 months	153	172
	1 year	110	123

KOOS-JR, knee injury and osteoarthritis outcome score for joint replacement; NRS, numeric rating scale.

## PRP Characterization

PRP characterization data were available for only 102 of 743 patients, limiting the ability to evaluate dose-response relationships. Platelet ratio values were derived from the final platelet concentration divided by baseline whole blood platelet count. The majority of patients lacked ratio data due to the difficulty of collecting, transporting, and analyzing samples from this high volume of patients.

Patients were evaluated longitudinally at baseline, 6 months, and 1 year after treatment. Clinical outcomes were assessed using the Pain NRS (scored on a 0-100 scale, with higher scores indicating greater pain intensity),^13^ the KOOS-JR,^25^ and the Patient Acceptable Symptom State (PASS).^41^ The primary outcomes of the present study were changes in pain and function over time, as measured by Pain NRS and KOOS-JR, as well as symptom state measured by PASS. Subgroup analyses were performed according to radiographic severity, stratified as KL grades 1 to 3 versus KL grade 4. In addition, the association between patient- and treatment-specific factors and clinical response was examined.

For longitudinal PROM analysis, evaluation was restricted to baseline, 6-month, and 1-year timepoints. Two analyses were performed for each PROM to assess the consistency of findings and the potential impact of incomplete follow-up on outcome estimates. The first was a complete-case analysis, which included 59 patients who completed all 3 timepoints for both PROMs. The second was an all-response analysis, which included all available responses for each PROM, resulting in different analytic cohorts for each outcome measure. Specifically, the all-response analysis included 605 Pain NRS observations from 391 patients, and 638 KOOS-JR observations from 400 patients. Model-based estimates were reported for each timepoint, together with 95% CIs, differences from baseline, standard errors, and *P* values. As these estimates were derived from fitted models, they may differ slightly from raw observed means. Sensitivity analyses showed that results from the complete-case analysis were generally consistent with those of the all-response analysis, suggesting that variation in PROM completion did not alter the all-response analysis findings meaningfully. The all-response analysis dataset was used for the primary analysis to maximize sample size and statistical power. Although 743 patients underwent PRP injection, the analyzed cohorts for each PROM were smaller due to incomplete follow-up. Specifically, 391 patients contributed Pain NRS data and 400 contributed KOOS-JR data, while only 59 patients completed both PROMs at all 3 timepoints. As such, the all-response analysis reflects all available observations rather than complete longitudinal follow-up for all participants.

The PASS was analyzed separately from the longitudinal PROM analysis using established thresholds for each outcome measure. PASS represents the threshold beyond which patients consider their symptoms as acceptable. PASS was defined as a Pain NRS score of <30 and a KOOS-JR score of ≥71. PASS rates were summarized at baseline, 6 months, and 1 year to determine the proportion of patients achieving an acceptable symptom state over time. Rates were evaluated for the overall cohort and stratified by radiographic severity (KL grades 1 to 3 vs KL grade 4). The percentages reported at each timepoint indicate the proportion of patients whose scores met the respective PASS cutoff. For example, a Pain NRS PASS rate of 80% indicates that 80% of patients reported pain levels <30, whereas a KOOS-JR PASS rate of 48% indicates that 48% of patients reported KOOS-JR scores ≥71.

## Statistical Analysis

Continuous variables were summarized as means with standard deviations or, when appropriate, as model-based estimates with 95% CIs. Categorical variables were reported as frequencies and percentages. Repeated-measures analysis of variance was used to assess changes in PROM scores over time. Separate repeated-measures analyses were performed for each PROM in the complete-case analysis cohort and all-response analysis cohort. Additional stratified analyses were performed by KL grade category. A *P* value < 0.05 was considered statistically significant. All analyses were performed using SAS software, Version 9.4 (SAS Institute, Inc).

## Results

A total of 743 patients undergoing PRP injections met the inclusion criteria and constituted the treated cohort. Of these, 405 patients contributed to ≥1 PROM analysis: 391 patients for Pain NRS and 400 patients for KOOS-JR, with time-specific sample sizes at baseline, 6 months, and 1 year reported in Table 1. Baseline demographic and clinical characteristics are presented in Tables 2 and 3.

**Table 2.** Demographic and clinical characteristics of the all-response analysis cohort (N = 743)

	All patients (n = 743)		Patients included in ≥1 PROM analysis (n = 405)	
	Mean	SD	Mean	SD
	62.63	13.89	63.49	12.77
Age	n	%	n	%
Sex				
Female	371	49.93	204	50.37
Male	372	50.07	201	49.63
Race				
Asian	23	3.10	18	4.44
Black or African American	15	2.02	6	1.48
Other or multiracial	42	5.65	22	5.43
Unknown	29	3.90	10	2.47
White	634	85.33	349	86.17
Ethnicity				
Hispanic or Latino	34	4.58	11	2.72
Not Hispanic or Latino	674	90.71	379	93.58
Unknown	35	4.71	15	3.70
Laterality				
Unilateral	516	69.45	290	71.60
Bilateral	227	30.55	115	28.40
Injection method				
Manual	431	58.01	285	70.37
US-guided	312	41.99	120	29.63
Platelet ratio				
Not available	641	86.27	303	74.81
0.3 to <1.8	46	6.19	46	11.36
1.8 to 7.8	56	7.54	56	13.83
	n	%		
KL grade*^a^*				
KL 1 to 3	487	65.55		
KL 4	256	34.45		

KL, Kellgren-Lawrence; PROM, patient-reported outcome measure; US, ultrasound.

a KL grade 1 to 3, 65.55%; grade 4, 34.45%

**Table 3.** Demographic and clinical characteristics for each subset of patients used in PROM regression models

	Pain NRS (n = 391)		KOOS-JR (n = 400)	
	Mean	SD	Mean	SD
	63.59	12.62	63.57	12.74
Age	n	%	n	%
Sex				
Female	199	50.90	202	50.50
Male	192	49.10	198	49.50
Race				
Asian	17	4.35	18	4.50
Black or African American	6	1.53	6	1.50
Other or multiracial	21	5.37	22	5.50
Unknown	9	2.30	10	2.50
White	338	86.45	344	86.00
Ethnicity				
Hispanic or Latino	11	2.81	11	2.75
Not Hispanic or Latino	366	93.61	374	93.50
Unknown	14	3.58	15	3.75
Laterality				
Unilateral	280	71.61	287	71.75
Bilateral	111	28.39	113	28.25
Injection method				
Manual	280	71.61	282	70.50
US-guided	111	28.39	118	29.50
Platelet ratio				
Not available	291	74.42	298	74.50
0.3 to <1.8	45	11.51	46	11.50
1.8 to 7.8	55	14.07	56	14.00

KOOS-JR, knee injury and osteoarthritis outcome score for joint replacement; NRS, numeric rating scale; PROM, patient-reported outcome measure; US, ultrasound.

Pain NRS analyses included 391 unique patients contributing 605 observations across the 3 timepoints; KOOS-JR analyses included 400 unique patients contributing 638 observations across the 3 timepoints. Per-timepoint counts represented in Table 1 reflect response availability at each visit and do not sum to these unique-patient totals, as individual patients could contribute to >1 timepoint.

### Primary Outcomes

#### Pain (Pain NRS)

Pain NRS improved over time in the all-response analysis cohort across all KL grades (1-4). The estimated mean Pain NRS score decreased from 39.2 at baseline to 27.2 at 6 months and 22.4 at 1 year (both *P* < 0.001) (Figure 1).

**Figure 1.**
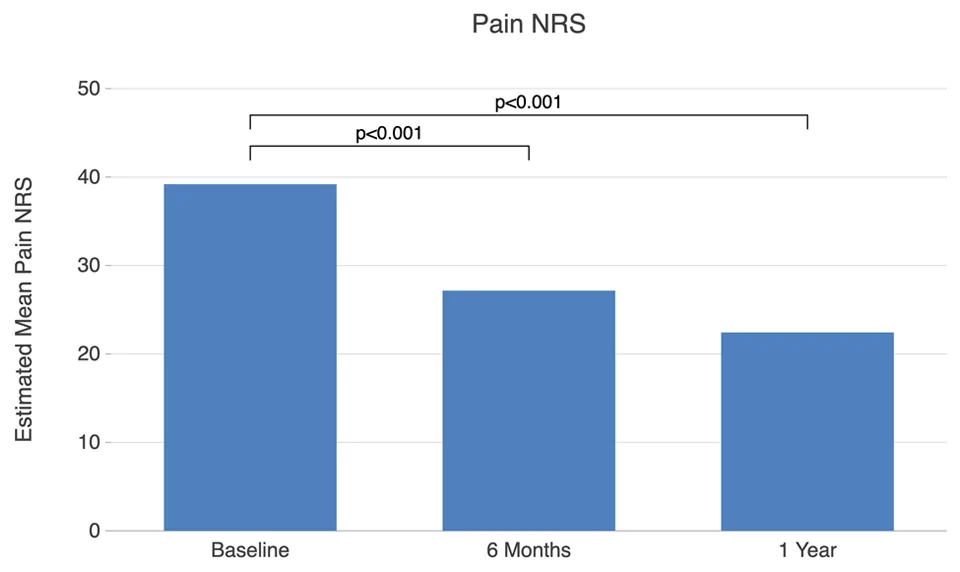
Regression model estimates of Pain NRS at baseline, 6 months, and 1 year in the all-response analysis cohort across KL grades 1 through 4. KL, Kellgren-Lawrence; NRS, numeric rating scale.

When stratified by radiographic severity, patients with KL grades 1 to 3 demonstrated a decrease in mean pain scores from 38.2 at baseline to 24.3 at 6 months and 22.7 at 1 year, respectively (*P* < 0.001). Patients with KL grade 4 also improved, with mean pain scores decreasing from 40.2 at baseline to 30.0 at 6 months (*P* = 0.007) and 22.2 at 1 year (*P* < 0.001) (Figure 2).

**Figure 2.**
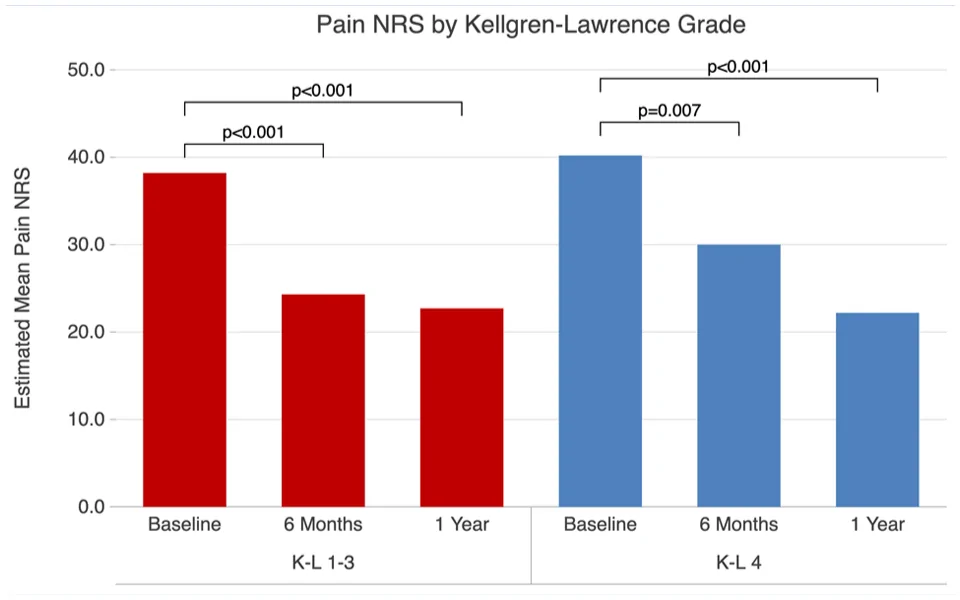
Pain NRS over time stratified by KL grade (KL 1 to 3 vs KL 4) in the all-response analysis cohort. KL, Kellgren-Lawrence; NRS, numeric rating scale.

#### Functional Outcomes (KOOS-JR)

Functional outcomes measured using the KOOS-JR, similarly improved over time in the all-response analysis cohort. The estimated mean KOOS-JR score increased from 60.7 at baseline to 68.5 at 6 months and 69.1 at 1 year (both *P* < 0.001) (Figure 3).

**Figure 3.**
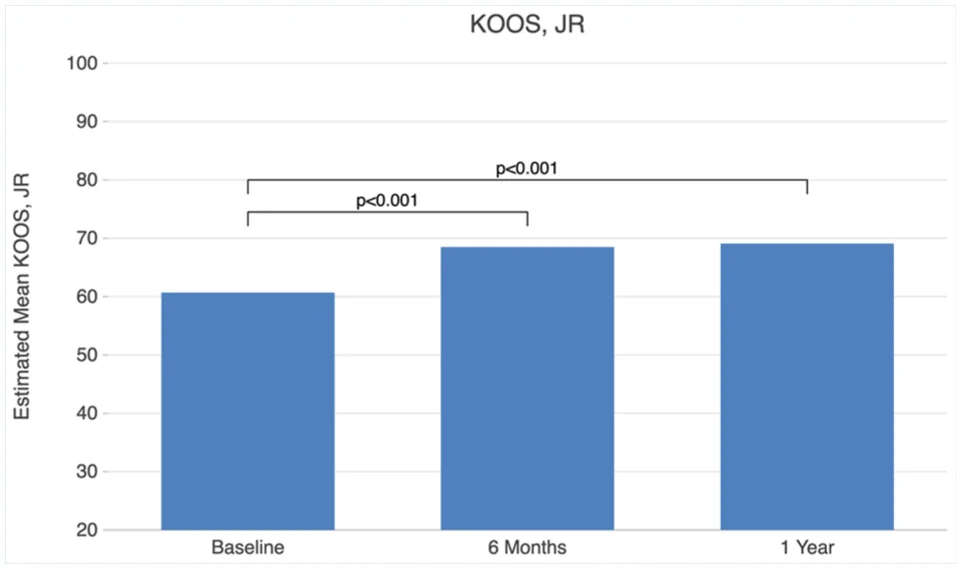
Regression model estimates of KOOS-JR at baseline, 6 months, and 1 year in the all-response analysis cohort across KL grades 1 through 4. KL, Kellgren-Lawrence; KOOS-JR, knee injury and osteoarthritis outcome score for joint replacement.

When stratified by osteoarthritis severity, patients with KL grades 1 to 3 demonstrated an increase in mean KOOS-JR scores from 61.9 at baseline to 72.7 at 6 months and 72.6 at 1 year (both *P* < 0.001). Patients with KL grade 4 also improved, with mean KOOS-JR scores increasing from 59.5 at baseline to 64.3 at 6 months (*P* = 0.01) and 65.7 at 1 year (*P* = 0.001) (Figure 4).

**Figure 4.**
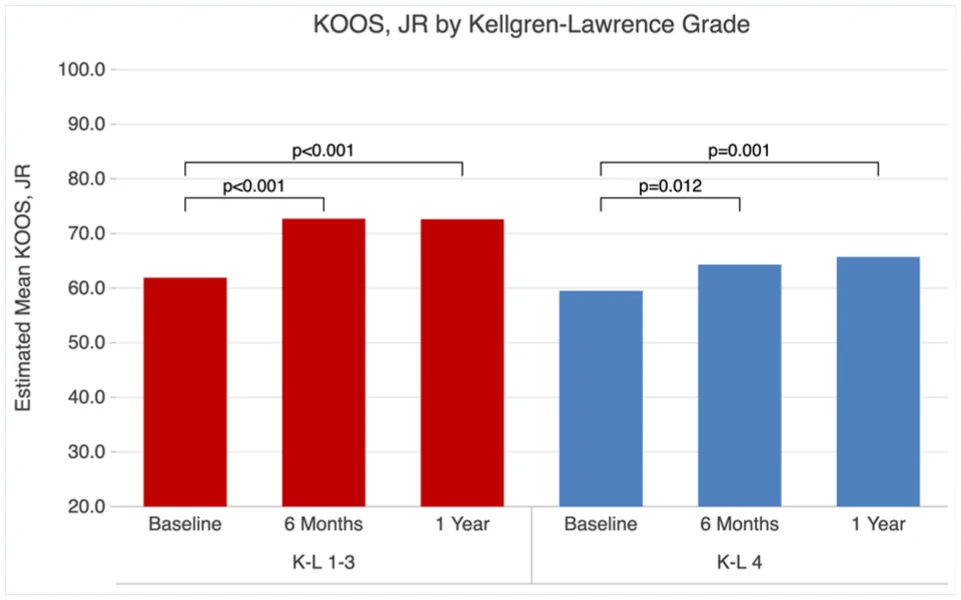
KOOS-JR over time stratified by KL grade (KL 1 to 3 vs KL 4) in the all-response analysis cohort. KL, Kellgren-Lawrence; KOOS-JR, knee injury and osteoarthritis outcome score for joint replacement.

#### Patient Acceptable Symptom State

The proportion of patients meeting the PASS for Pain NRS also increased over time, from 45% at baseline to 70% at 6 months and 80% at 1 year (Figure 5). Among patients with KL grades 1 to 3, the proportion of patients achieving PASS increased from 46% at baseline to 74% at 6 months and 71% at 1 year. Among patients with KL grade 4 disease, PASS achievement increased from 42% at baseline to 62% at 6 months and 78% at 1 year.

**Figure 5.**
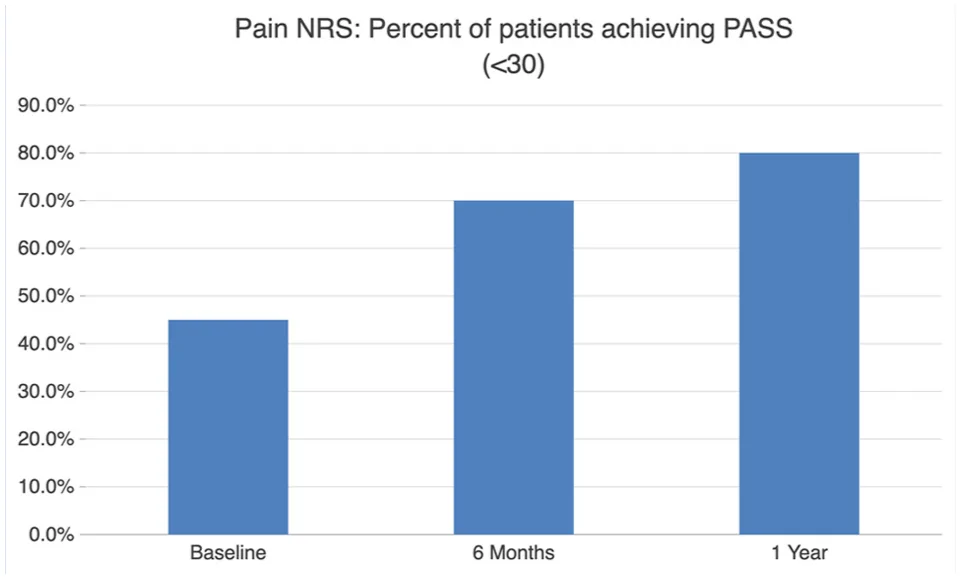
Proportion of patients with KL grades 1 to 4 achieving PASS over time for Pain NRS outcome measures. KL, Kellgren-Lawrence; NRS, numeric rating scale; PASS, patient acceptable symptom state.

In the cohort of patients with KL grades 1 to 4, PASS achievement for KOOS-JR increased from 18% at baseline to 47% at 6 months, with a rate of 41% at 1 year (Figure 6). Among patients with KL grades 1 to 3, PASS increased from 18% at baseline to 51% at 6 months and 49% at 1 year. Among patients with KL grade 4 disease, PASS increased from 18% at baseline to 41% at 6 months and 32% at 1 year.

**Figure 6.**
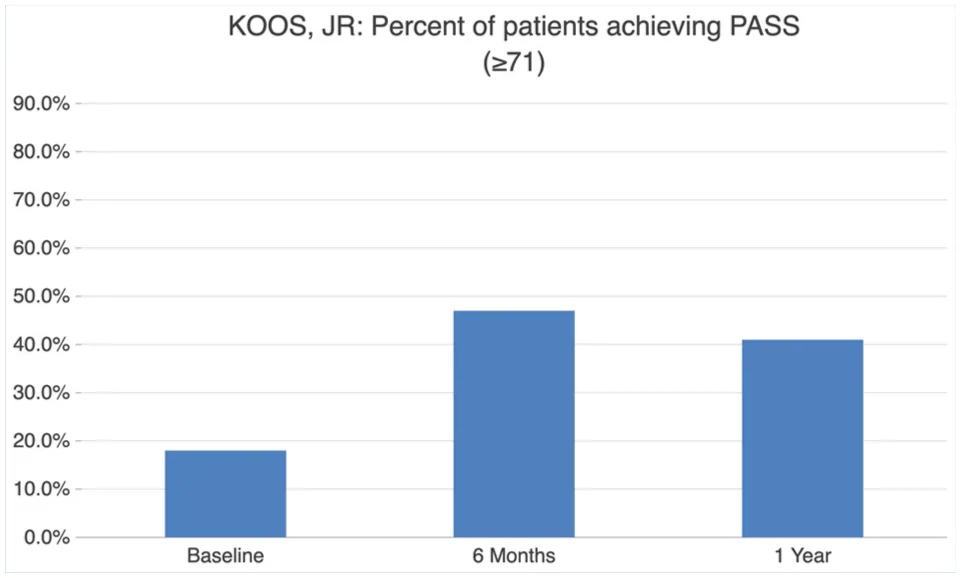
Proportion of patients with KL grades 1 to 4 achieving PASS over time for KOOS-JR outcome measures. KL, Kellgren-Lawrence; KOOS-JR, knee injury and osteoarthritis outcome score for joint replacement; PASS, patient acceptable symptom state.

### Subgroup Analysis

#### Platelet Concentration

Descriptive analyses and regression modeling were performed using the revised platelet ratio categorization of <1.8 versus ≥1.8. Platelet ratios were concentrated between approximately 1.5 and 2.3, with fewer observations at higher values, consistent with a right-skewed distribution.

As illustrated in Figure 7, outcome trajectories within the all-response analysis cohort differed by platelet ratio subgroup. The <1.8 group demonstrated reductions in Pain NRS at both 6 months and 1 year (both *P* < 0.001), whereas the ≥1.8 group did not show meaningful pain improvement (Figure 7a). In contrast, KOOS-JR improved meaningfully in both groups at 6 months and 1 year (Figure 7b). These findings suggest that lower platelet ratio values may be associated with greater pain responsiveness, whereas functional improvement appeared to occur regardless of platelet concentration.

**Figure 7.**
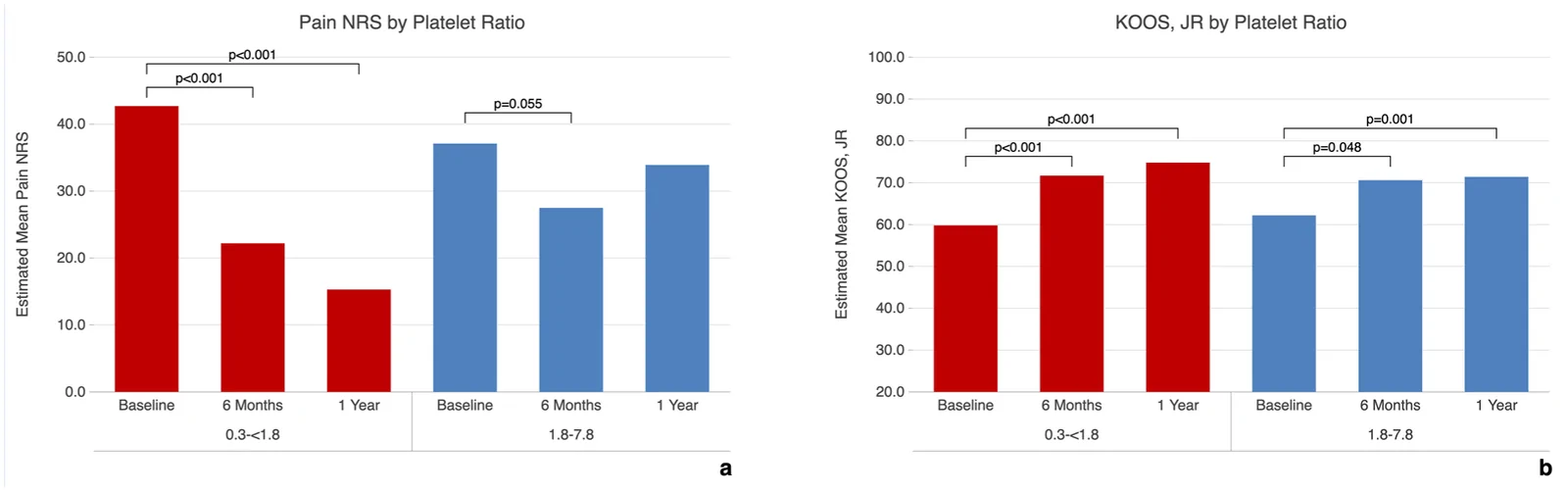
Regression model estimates of Pain NRS and KOOS-JR over time in the all-response analysis cohort, stratified by platelet ratio (<1.8 vs ≥1.8). (a) Pain NRS by platelet ratio. (b) KOOS-JR by platelet ratio. Significant within-group changes from baseline are shown with corresponding *P* values. KOOS-JR, knee injury and osteoarthritis outcome score for joint replacement; NRS, numeric rating scale.

#### US Versus Non-US

In subgroup analyses within the all-response analysis cohort stratified by injection method, both US-guided and landmark-guided intra-articular PRP injections were associated with improvement over time. For Pain NRS, estimated scores decreased from 41.8 to 24.4 at 6 months and 25.6 at 1 year in the US-guided group and from 37.8 to 26.7 at 6 months and 21.1 at 1 year in the landmark-guided group. KOOS-JR similarly improved in both groups, increasing from 58.8 to 68.2 and 67.8 in the US-guided group, and from 61.9 to 70.0 and 70.4 in the landmark-guided group at 6 months and 1 year, respectively.

Clinically meaningful improvements in Pain NRS and KOOS-JR were observed at both 6 months and 1 year for both injection methods. The magnitude and direction of change in pain and functional outcomes were broadly similar between US-guided and landmark-guided injections. As these analyses reflect within-group change over time rather than formal between-group comparisons, no definitive conclusions regarding the superiority of either injection method can be drawn from these data alone.

## Discussion

The principal finding of this study is that intra-articular PRP injections were associated with clinically meaningful improvements in pain, patient-reported function, and perceived symptom state in patients with knee OA over a 1-year follow-up period. Improvements were observed across all radiographic severity grades, with a greater magnitude of improvement among patients with less advanced disease. Importantly, a growing proportion of patients achieved an acceptable symptom state over time, indicating that observed improvements were meaningful from the patient’s perspective.

Improvement from baseline to 1 year was observed across all outcome measures in the all-response analysis cohort. Pain NRS improved from 39.2 at baseline to 27.2 at 6 months and 22.4 at 1 year, and KOOS-JR improved from 60.7 to 68.5 and 69.1. The proportion of patients achieving PASS - a threshold reflecting whether patients consider their symptoms acceptable in daily life - increased over time for Pain NRS and KOOS-JR measures. This finding suggests that a growing proportion of patients experienced improvement that was not only statistically measurable but also clinically relevant from the patient’s perspective. Pain outcomes demonstrated the greatest magnitude of improvement, with PASS rates increasing from 45% at baseline to 70% at 6 months and 80% at 1 year. Together, these findings describe clinically meaningful improvement in pain and symptom acceptability in this large cohort of patients with knee OA after PRP treatment.^4,12,16,29^ Because this study lacked a control group, these improvements cannot be attributed specifically to a biologic effect of PRP.

Knee OA is a progressive degenerative joint disease and a major cause of pain, functional limitation, and disability worldwide.^11,19,28,30^ Because OA is characterized by progressive structural and biochemical changes, there is substantial interest in nonsurgical treatment strategies that may improve symptoms and potentially delay progression to more invasive intervention.^3,19,23,27,28^ PRP has emerged as one such biologic option because of its potential anti-inflammatory and reparative effects through the delivery of growth factors and other bioactive mediators,^1,17,18,26,34,35^ although its clinical role remains debated because of heterogeneity in patient selection, injection protocols, and outcome assessment.^4,5,9,12,15,16,24,29,32,33^

The present findings are consistent with some previous randomized controlled trials, retrospective studies, and meta-analyses reporting that PRP may reduce pain and improve function in patients with knee OA, particularly in the short to intermediate term.^4,12,14,16,29,31,38^ Because this study did not include a control group, the observed improvements cannot be distinguished from placebo response, regression to the mean, or natural symptom fluctuation. Previous randomized controlled trials using leukocyte-poor PRP - including the RESTORE trial - have demonstrated no significant differences between PRP and saline placebo, with both groups showing improvement in pain scores over time. Therefore, the symptomatic improvements observed in the present cohort should be interpreted cautiously, as they may not reflect a specific biologic effect of PRP. In the present study, pain appeared to be the most responsive outcome domain, with the largest improvement observed in Pain NRS and the highest PASS achievement rates over time. KOOS-JR also demonstrated consistent improvement, with nearly half of all patients meeting PASS criteria at 6 months and 41% at 1 year. The observed KOOS-JR improvement of 8.4 points meets commonly cited MCID thresholds of approximately 7 to 10 points. Pain NRS in this registry was recorded on a scale of 0 to 100; on this scale, published minimal clinically important difference estimates are approximately 15 to 20 points, rather than the 1.5- to 2-point threshold that applies to the standard 0 to 10 NRS. The observed improvement in Pain NRS (39.2 to 22.4 at 1 year, a change of 16.8 points) meets this threshold, suggesting that the change was not only statistically significant, but also clinically meaningful. From a clinical perspective, the PASS analysis is particularly useful because it reflects whether patients reached a symptom level they considered acceptable in daily life, rather than only whether a score changed statistically. The consistent increase in PASS rates across both measures supports the clinical relevance of the observed PROM improvements.

Outcomes were also evaluated according to radiographic severity. Patients with KL grades 1 to 3 demonstrated improvement in Pain NRS and KOOS-JR at both 6 months and 1 year, and patients with KL grade 4 similarly improved, although the magnitude and consistency of response were somewhat more variable. PASS findings generally paralleled the PROM results. For KOOS-JR, patients with KL grades 1 to 3 had higher PASS achievement rates at follow-up than patients with KL grade 4, suggesting more consistent functional improvement in those with less advanced radiographic disease. For Pain NRS, PASS rates improved substantially in both severity groups, with high rates of symptom acceptability at 1 year, even among patients with KL grade 4 disease. These findings suggest that improvement in pain and function was observed across a broad range of radiographic disease severities after PRP injection, although patients with less advanced OA may experience more consistent improvement in function, knee symptoms, and global knee perception.

These results are noteworthy because previous literature has often suggested greater benefit of PRP in earlier stages of OA. Annaniemi et al^2^ reported that, although PRP injections resulted in significant improvements in pain and function across KL grades 1 to 3, patients with more advanced OA experienced smaller improvements and overall worse outcomes compared with those with mild disease. Similarly, Tripathi et al^40^ found that, although patients with early OA experienced significant pain reduction, those with advanced disease showed improvement primarily in stiffness, with less substantial pain relief than patients with earlier-stage disease. In contrast, the present investigation demonstrated that even patients with KL grade 4 disease experienced clinically meaningful improvement in pain and function over time. Although patients with less severe OA tended to show more consistent improvement across outcome domains, the present findings suggest that advanced radiographic disease does not necessarily preclude symptomatic improvement after PRP injection.

Growing evidence also suggests that patient-specific and treatment-specific factors may influence response to PRP.^5,9,33,36,39,42^ Previous studies have shown that age, sex, PRP composition, platelet concentration, and treatment dosing may contribute to variability in outcomes.^6-8,10,20,32,36,39,42^ In the present cohort, patients with similar radiographic severity still demonstrated substantial variability in symptoms and function, which is consistent with previous work showing that physical function is influenced not only by structural severity but also by pain and other patient-level factors.^37^ Sun et al^38^ similarly reported that, although cartilage repair capacity may decline with age, the clinical effectiveness of PRP may depend more on local biologic activity and inflammatory regulation than on age alone - a finding consistent with previous studies suggesting that PRP may retain therapeutic benefit across age groups.^10,20^

The interpretation of PRP studies remains complicated by the lack of standardization in biologic preparation and delivery. Previous investigations have used heterogeneous PRP formulations with variation in platelet concentration, leukocyte composition, injection frequency, and adjunctive treatment protocols, making direct comparison across studies difficult.^5,9,15,16,24,32,33^ Such heterogeneity limits the ability to make strong recommendations regarding the optimal PRP product or treatment protocol.^5,9,15,24,33^ In the present study, all injections utilized leukocyte-poor PRP preparations, which enhanced internal consistency, although variability in other procedural factors remained reflective of real-world clinical practice. Recent literature has suggested that greater platelet concentration or platelet dose may be associated with greater clinical improvement. Bensa et al^6^ reported that higher platelet concentration PRP was associated with clinically significant improvement in pain and function. Similarly, Boffa et al^8^ demonstrated a positive correlation between platelet concentration and clinical outcomes, and Berrigan et al^7^ concluded that a greater platelet dose may yield better clinical outcomes in knee OA. The finding that patients with platelet ratios <1.8 demonstrated greater pain improvement than those with ratios ≥1.8 contradicts previous literature suggesting higher platelet concentrations may be associated with greater clinical benefit. A clear dose-response relationship for platelet concentration was not established in the present study, highlighting the need for greater standardization in future investigations. The platelet ratio distribution demonstrated a concentration of values in the lower range, with relatively few patients at higher ratios. This skewed distribution suggests that the 1.8 cutoff may be useful for group-based comparisons, although the sparse observations at the higher end may limit statistical precision, and these findings warrant confirmation in a larger and more evenly distributed cohort.

In addition to radiographic severity, the present study explored whether US guidance was associated with clinical outcomes. No clinically meaningful differences were observed between US-guided and landmark-guided intra-articular injections with respect to pain, function, or symptom acceptability at follow-up, suggesting that the guidance method did not materially influence clinical response in this cohort. This may reflect that both techniques were performed by experienced sports medicine physicians in a high-volume specialty setting. Although US guidance may improve procedural accuracy, the present findings suggest that its effect on short- to intermediate-term patient-reported outcomes after intra-articular PRP injection may be limited.

Guideline recommendations regarding PRP for knee OA remain conflicting. The American College of Rheumatology strongly recommends against PRP for knee OA based on available evidence, whereas the American Academy of Orthopaedic Surgeons provides a limited recommendation suggesting PRP may reduce pain and improve function. The present findings should be interpreted within this broader context, and clinical use of PRP should be individualized.

The present investigation also used both the all-response analysis and the complete-case analysis for longitudinal assessment, which represents an additional methodological strength. Because PROM completion varied across timepoints, sensitivity analyses were performed in the subset of 59 patients who completed both PROMs at baseline, 6 months, and 1 year. Findings from the complete-case analyses were generally consistent in direction and magnitude with those of the all-response analyses; however, given the small complete-case sample (n = 59), these findings should be considered hypothesis-generating rather than confirmatory of robustness to incomplete follow-up. In the complete-case analysis cohort, pain NRS improved from 41.4 at baseline to 27.1 at 6 months (*P* = 0.001) and 20.1 at 1 year (*P* < 0.001). KOOS-JR improved from 59.8 at baseline to 70.6 at 6 months and 71.3 at 1 year (both *P* < 0.001). The use of multiple outcome measures also strengthens the study, as Pain NRS and KOOS-JR allowed independent assessment of pain and function, while PASS provided a clinically interpretable measure of symptom acceptability from the patient’s perspective.

The present study has limitations. This investigation was a retrospective cohort study without a control group; therefore, causality cannot be established. Follow-up completion decreased over time, which may have introduced selection bias and reduced statistical power at later timepoints. The substantial attrition observed in this study introduces risk of bias, as patients who continued to complete PROMs may differ systematically from those lost to follow-up. This surviving sample may overestimate treatment benefit, and the results should be interpreted with caution. Formal comparison of baseline characteristics between patients retained in follow-up and those lost to follow-up was not performed, as KL grade and other radiographic data were reported only for the overall treated cohort (Table 2) rather than stratified by follow-up status. The extent to which retained and lost patients differed therefore cannot be determined from the present data, and unmeasured differences may remain. Although the all-response analysis allowed inclusion of all available data and the complete-case analysis showed similar overall trends, patients who remained engaged in follow-up may represent a systematically different population from those who did not complete outcome measures. The study also relied on patient-reported outcomes without routine follow-up imaging, which limits assessment of structural joint changes after treatment. PASS thresholds for KOOS-JR were derived originally from post total knee arthroplasty populations and have not been validated for nonsurgical management of knee OA. This represents a limitation of the present analysis. Concurrent treatments such as physical therapy, oral medications, or additional injections were not collected systematically and may represent confounders influencing symptom trajectories. Because the registry did not systematically document individual adverse events, safety outcomes could not be assessed reliably in this study. This represents a limitation of the present analysis, and future prospective studies of PRP for knee OA should incorporate standardized adverse event tracking. Finally, treatment decisions and procedural details reflected real-world clinical practice, which enhances generalizability but also introduces heterogeneity - an issue described commonly in PRP literature.^9,33^

Conversely, the present study includes a large real-world cohort of patients with knee OA treated within a single institutional registry and provides longitudinal evaluation of multiple validated PROMs. The consistency of findings across both the all-response analysis and complete-case analysis strengthens the interpretation of the results.

These data describe the clinical course of patients with knee OA after intra-articular PRP treatment and are consistent with previous systematic reviews and clinical studies reporting pain relief and improved symptom acceptability after PRP; because this study lacked a control group, however, these findings should not be interpreted as establishing that PRP caused the observed improvements.^4,12,16,29^ Future prospective studies with standardized treatment protocols, active comparison groups, and improved follow-up rates are needed to better define predictors of response and the role of PRP in the management of knee OA.^5,9,33^

## Conclusion

Intra-articular PRP injections were associated with improvement in pain, patient-reported function, and symptom state in patients with knee OA over a 1-year follow-up period. In the primary all-response analysis, statistically significant improvement from baseline to 1 year was observed in Pain NRS and KOOS-JR across a broad spectrum of radiographic disease severity. A growing proportion of patients achieved PASS over time, further supporting the clinical relevance of these findings. Although these findings suggest that symptomatic improvement occurred in this patient population after PRP injection, this study represents a retrospective analysis without a control group; therefore, definitive treatment recommendations cannot be made. Further prospective, controlled studies with standardized treatment protocols, active comparison groups, longer follow-up, and more complete outcome assessment are needed to better define the role of PRP in the management of knee OA and identify predictors of treatment response.

## Clinical Recommendations

1. Intra-articular PRP injections may be considered as a treatment option for patients with symptomatic knee OA who have not achieved adequate relief with conservative management, with the understanding that this study cannot establish a causal treatment effect. Strength of Recommendation: C
2. Improvement in patient-reported pain and function after PRP injection was observed across a broad range of radiographic disease severities, including patients with KL grade 4 disease, although patients with less advanced osteoarthritis (KL grades 1-3) tended to demonstrate more consistent improvement. Strength of Recommendation: C
3. Clinicians should inform patients regarding the variability in PRP preparation methods, differences in treatment protocols, and the current absence of consensus standardization across studies when discussing treatment expectations. Strength of Recommendation: C
